# Tobacco smoke carcinogens exacerbate APOBEC mutagenesis and carcinogenesis

**DOI:** 10.21203/rs.3.rs-5843684/v1

**Published:** 2025-06-03

**Authors:** Cameron Durfee, Erik N. Bergstrom, Marcos Díaz-Gay, Yufan Zhou, Nuri Alpay Temiz, Mahmoud A. Ibrahim, Shuvro P. Nandi, Yaxi Wang, Xingyu Liu, Christopher D. Steele, Joshua Proehl, Rachel I. Vogel, Prokopios P. Argyris, Ludmil B. Alexandrov, Reuben S. Harris

**Affiliations:** 1 Department of Biochemistry and Structural Biology, University of Texas Health San Antonio, San Antonio, Texas, USA, 78229; 2 Department of Cellular and Molecular Medicine, University of California San Diego, La Jolla, California, USA, 92093; 3 Department of Bioengineering, University of California San Diego, La Jolla, California, USA, 92093; 4 Moores Cancer Center, University of California San Diego, La Jolla, California, USA, 92093; 5 Digital Genomics Group, Structural Biology Program, Spanish National Cancer Research Center (CNIO), Madrid, Spain, 28029; 6 Institute for Health Informatics, University of Minnesota, Minneapolis, Minnesota, USA, 55455; 7 Masonic Cancer Center, University of Minnesota, Minneapolis, Minnesota, USA, 55455; 8 Department of Obstetrics, Gynecology, and Women’s Health, University of Minnesota, Minneapolis, Minnesota, USA, 55455; 9 Division of Oral and Maxillofacial Pathology, College of Dentistry, Ohio State University, Columbus, Ohio, USA, 43210; 10 Howard Hughes Medical Institute, University of Texas Health San Antonio, San Antonio, Texas, USA, 78229

**Keywords:** APOBEC mutagenesis, bulky DNA adducts, cancer development and progression, nucleotide excision repair, tobacco carcinogens

## Abstract

Mutations in somatic cells are inflicted by both extrinsic and intrinsic sources and contribute over time to cancer. Tobacco smoke contains chemical carcinogens that have been causatively implicated with cancers of the lung and head & neck^1,2^. APOBEC family DNA cytosine deaminases have emerged as endogenous sources of mutation in cancer, with hallmark mutational signatures (SBS2/SBS13) that often co-occur in tumors of tobacco smokers with an equally diagnostic mutational signature (SBS4)^3,4^. Here we challenge the dogma that mutational processes are thought to occur independently and with additive impact by showing that 4-nitroquinoline 1-oxide (NQO), a model carcinogen for tobacco exposure, sensitizes cells to APOBEC3B (A3B) mutagenesis and leads to synergistic increases in both SBS2 mutation loads and oral carcinomas *in vivo*. NQO-exposed/A3B-expressing animals exhibit twice as many head & neck lesions as carcinogen-exposed wildtype animals. This increase in carcinogenesis is accompanied by a synergistic increase in mutations from APOBEC signature SBS2, but not from NQO signature SBS4. Interestingly, a large proportion of A3B-catalyzed SBS2 mutations occurs as strand-coordinated pairs within 32 nucleotides of each other in transcribed regions, suggesting a mechanism in which removal of NQO-DNA adducts by nucleotide excision repair exposes short single-stranded DNA tracts to enzymatic deamination. These highly enriched pairs of APOBEC signature mutations are termed *didyma* (Greek for twins) and are mechanistically distinct from other types of clustered mutation (*omikli* and *kataegis*). Computational analyses of lung and head & neck tumor genomes show that both APOBEC mutagenesis and *didyma* are elevated in cancers from smokers compared to non-smokers. APOBEC signature mutations and *didyma* are also elevated in normal lung tissues in smokers prior to cancer initiation. Collectively, these results indicate that DNA adducting mutagens in tobacco smoke can amplify DNA damage and mutagenesis by endogenous APOBEC enzymes and, more broadly, suggest that mutational mechanisms can interact synergistically in both cancer initiation and promotion.

Over the past decade, studies on somatic mutagenesis have been revolutionized by significant advancements in DNA sequencing technologies and computational methods for analyzing large-scale genomics datasets. These advancements have enabled the mapping of patterns of somatic mutations imprinted by different mutational processes, termed *mutational signatures*. Nearly 100 distinct single base substitution (SBS) mutational signatures have been described in human cancers, with approximately half assigned to putative mechanistic etiologies, which can be categorized broadly as arising from either endogenous cellular processes or exogenous environmental factors^3,5,6^. Major endogenous sources include unavoidable chemical reactions such as spontaneous deamination of 5-methyl-cytosine to thymine (C-to-T) in SBS1^7–9^ and oxidation of guanine to 8-oxoG leading to G-to-T transversions (C-to-A in SBS18)^10^, as well as defects in normal DNA repair processes such as homologous recombination (broad mutational spectrum in SBS3 as well as larger-scale genomic aberrations)^8,11,12^. Significant exogenous sources include mutagens in tobacco smoke, ultraviolet (UV) light, and aristolochic acid^3^. Tobacco smoke has many chemical mutagens but the most potent classes are polycyclic aromatic hydrocarbons (such as benzo[*a*]pyrene) and tobacco-specific nitrosamines (such as *N’*-nitrosonornicotine)^2^. Upon metabolic activation, both classes predominantly form adducts with guanine nucleobases and to lesser extents adenines and thymines, leading to the major G-to-T (C-to-A) mutational contribution in SBS4^13,14^. UV light cross-links adjacent pyrimidine bases, which results in error-prone lesion bypass synthesis (A-insertion) by DNA polymerases and C-to-T mutations (SBS7a/b)^15^. Aristolochic acid is processed into DNA reactive compounds that lead to A-to-T transversions (SBS22)^15^. Many other sources of DNA damage also contribute to human mutational signatures but these are less common. Mutational processes in general, as well as in cancer, are assumed to occur independently of one another regardless of etiology, with additive impact throughout the lifespan of an individual^9,16,17^.

Cellular APOBEC3 enzymes, predominantly APOBEC3B (A3B) and APOBEC3A (A3A)^18–21^, generate SBS2 and SBS13 mutational signatures and represent the second most common mutational process in human cancer (following aging-associated processes; *viz.*, SBS1 and SBS5)^3^. A3B and A3A normally function alongside other family members in innate antiviral immunity^22^. However, it is now clear that approximately 70% of all cancer types are impacted by their mutagenic activity, especially tumors of the bladder, breast, cervix, head & neck, and lung tissues^3,8,23–26^. A3B and A3A preferentially catalyze the hydrolytic deamination of cytosine-to-uracil in TCA and TCT motifs in single-stranded (ss)DNA^27–29^. The resulting uracil lesions can either template the insertion of adenines during DNA replication (leading to C-to-T in SBS2) or, be excised by uracil DNA glycosylase and converted into abasic sites. Abasic sites can also mis-template the insertion of adenines during DNA replication (also leading to C-to-T in SBS2) or provoke lesion by-pass synthesis by the deoxy-cytitidyl transferase REV1 (leading to C-to-G and C-to-A in SBS13)^19^. Additional processing of abasic sites can result in single- and double-stranded DNA breaks and consequently other types of mutations including insertion/deletion mutations (indels) and larger-scale structural variations such as translocations^18,20,30^. Most APOBEC3 signature mutations in cancer are dispersed and associated with DNA replication, transcription (R-loops), and recombination intermediates^31–36^. However, APOBEC3 mutations also occur in clusters called *omikli* (2–3 mutations) associated with DNA mismatch repair and *kataegis* (≥4 mutations) associated with sites of chromosomal DNA breakage, R-loop accumulation, and extrachromosomal DNA formation^8,31,35,37^. Although APOBEC3 enzymes are now a well-established source of mutation in cancer, potential interactions with other sources of DNA damage and mutation have not been investigated.

## Exogenous NQO and endogenous A3B exhibit carcinogenic synergy

**Figure 1.**
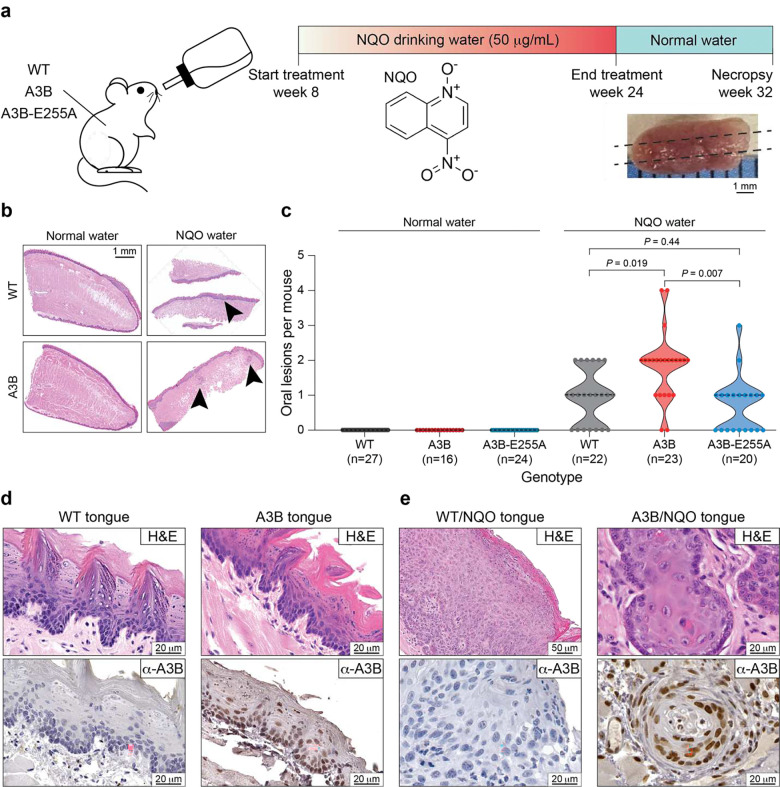
Head & neck tumor formation *in vivo* through A3B and NQO mutagenesis. (**a**) Schematic of the NQO treatment procedure. Animals are treated with NQO for 16 weeks, provided with normal water for 8 weeks, and then sacrificed for analysis including longitudinal sectioning of the tongue and histopathology. (**b**) Representative H&E stained tongue tissues from WT (top) and A3B (bottom) mice after receiving normal water (left) or the NQO procedure (right). The arrowhead in the top right points to an area of epithelial dysplasia, and the arrowheads in the bottom right indicate foci of invasive squamous cell carcinoma (SCC) (scale = 1 mm). (**c**) Quantification of oral lesions in NQO-treated animals (right) in comparison to historic controls provided with normal water (left). Each dot is quantification from an independent animal, and dotted lines represent median tumor numbers (*P*-values, Poisson regression). (**d**,**e**) H&E (top) and anti-A3B (bottom) stained tongue tissues from WT and A3B mice after receiving normal water (left) or the NQO procedure (right) (scale bars indicated). The lingual surface epithelium in panel d has no evidence of cytologic atypia. Nuclear A3B staining is strong in basal and spinous cells. Images in panel e are higher magnifications of lesions shown in panel b. The left-side H&E-stained photomicrograph in panel e demonstrates epithelial dysplastic aberrations including maturational disorganization, precocious keratinization and increased nuclear-to-cytoplasmic ratio. The right-side H&E-stained photomicrograph depicts invasive SCC comprising islands and nests of malignant epithelial cells featuring enlarged, hyperchromatic nuclei with macronucleoli infiltrating the fibrous stroma.

Human head & neck cancer is commonly modeled in mice by administering the tobacco carcinogen mimetic 4-nitroquinoline 1-oxide (NQO) in drinking water, which results in visible oral tumors within 16–24 weeks^38,39^. Because mutational signatures from tobacco smoke and APOBEC enzymes often occur in the same head & neck and lung tumors^40^, here we sought to test for possible mutagenic interactions between NQO and A3B. This was done by applying the established NQO mutagenesis procedure to wildtype (WT), human A3B, and human A3B-E255A (catalytic mutant) expressing C57BL/6 animals (workflow in Fig. 1a). Human A3B protein levels in these animals (*Rosa26::CAG-L-A3Bi*), as shown by our prior studies^30^, approximate those observed in many human head & neck and lung cancers and result in accelerated tumorigenesis dependent on catalytic activity (78 week average penetrance in A3B animals vs 100 weeks in WT animals and 92 weeks in A3B-E255A animals). Tissue specimens and sequencing data from these prior experiments are used here for comparison. As anticipated from such long tumor latencies with normal drinking water and SPF housing conditions, no oral lesions were evident at the 32 week timepoint for A3B, A3B-E255A, or WT animals (representative histology, lesion numbers, and images in Fig. 1b,c,d). Additional A3B expressing animals were sacrificed at 32 weeks of age to re-confirm that no tumor formation is evident at this early timepoint without NQO treatment.

**Extended Data Figure 1.**
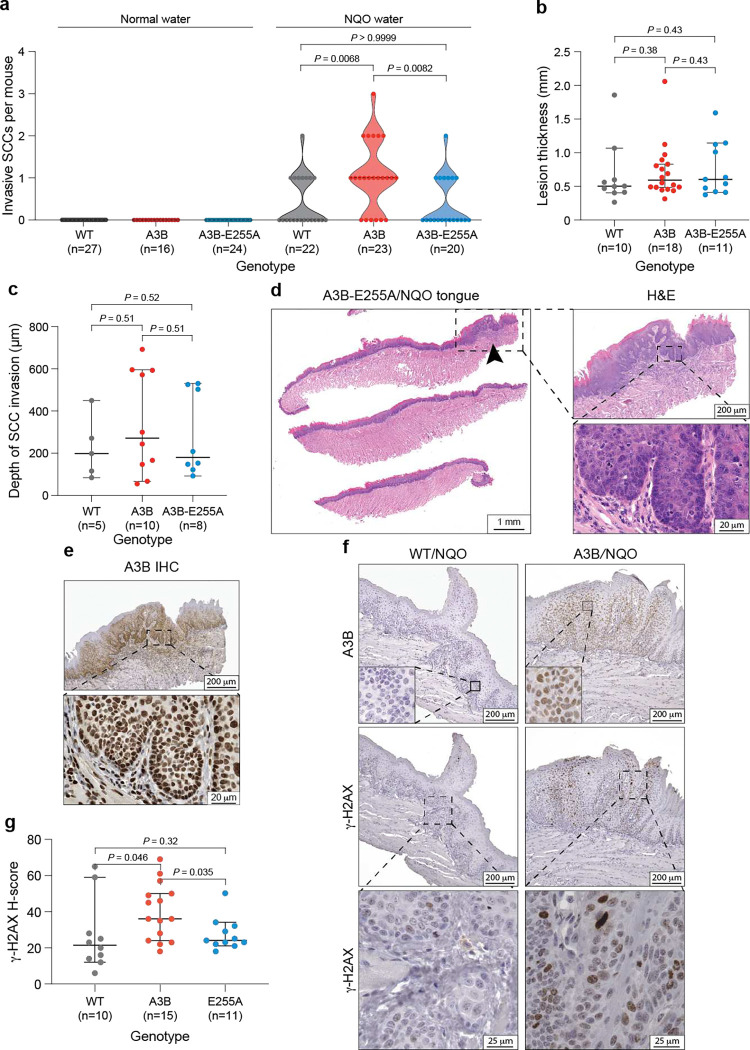
Additional histopathology of oral lesions. (**a**) Quantification of oral invasive SCCs in NQO-treated animals (right) in comparison to historic controls provided with normal water (left). Each dot represents SCC quantification from an independent animal, and the dotted lines represent medians (*P*-values, pairwise Mann-Whitney U tests). (**b**) Quantification of lesion thickness of the exophytic papillary high-grade epithelial dysplasias that developed in the oral cavity of animals with indicated genotypes. The horizontal lines and whiskers represent medians and 95% confidence intervals (*P*-values, pairwise Mann-Whitney U tests). (**c**) Quantification of the depth of invasion of each invasive squamous cell carcinoma that developed in the oral cavity of animals with indicated genotypes. The horizontal lines and whiskers represent medians and 95% confidence intervals (*P*-values, pairwise Mann-Whitney U tests). (**d**) Representative H&E staining of a tongue from an E255A-A3B mouse, with an arrow indicating an area of high-grade epithelial dysplasia. The dysplastic area is enlarged 5x and 50x to the right, with scale bars indicated. (**e**) A3B-E255A staining of an adjacent section of the tongue lesion shown in panel d. (**f**) Representative IHC images of high-grade epithelial dysplasias from WT (left) and A3B (right) mice treated with NQO. Lesions from WT animals stain negative for A3B and γ-H2AX, whereas those from A3B animals stain strongly for both of these proteins. Nuclear A3B is most evident in the top right inset image and γ-H2AX in the bottom right panel. (**g**) H-score quantification of γ-H2AX staining of high-grade oral epithelial dysplasias from mice with the indicated genotypes. The horizontal lines and whiskers represent medians and 95% confidence intervals (*P*-values, pairwise Mann-Whitney U tests).

In contrast, NQO-treated WT mice exhibit an average of 1 oral mucosal lesion per animal at the 32 week experimental endpoint (representative histology in Fig. 1b, lesion numbers in Fig. 1c, and representative images in Fig. 1e). Oral mucosal lesions are defined here as clinically or microscopically distinct exophytic papillary dysplastic lesions or invasive squamous cell carcinomas (SCCs) (combined data from 2 sets of 3 physically separated 4 μm thin sections from each animal’s tongue plus oral cavity; Methods). Interestingly, twice as many lesions are evident in the NQO-treated A3B-expressing group, where these mice develop an average of 2 and as many as 4 lesions per animal (representative histology in Fig. 1b, lesion numbers in Fig. 1c, and representative images in Fig. 1e). This synergistic effect requires the deaminase activity of A3B, because otherwise isogenic A3B catalytic mutant animals (A3B-E255A) treated with NQO exhibit lesion numbers indistinguishable from NQO-treated WT animals (Fig. 1c). A3B catalytic activity also affects the malignant potential of lesions, as evidenced by increased numbers of invasive SCCs in the tongues of A3B/NQO animals but not in A3B-E255A/NQO counterparts (Extended Data Fig. 1a). However, A3B does not appear to affect individual tumor thickness or the depth of carcinoma invasion (Extended Data Fig. 1b,c). Immunohistochemistry (IHC) with a monoclonal antibody that recognizes human A3B^41^ confirms that WT mice lack A3B, whereas both catalytically active and inactive A3B animals express this protein at similar levels in the nuclear compartment of histologically normal tongue epithelia as well as in oral epithelial dysplastic lesions and invasive SCCs (Fig. 1d,e; Extended Data Fig. 1d,e,f). Interestingly, tumors from A3B/NQO mice also manifest a DNA damage response at the 32 week experimental endpoint (8 weeks after NQO withdrawal) as indicated by higher γ-H2AX levels (Extended Data Fig. 1f,g).

### NQO sensitizes the genome to mutagenesis by A3B

**Extended Data Figure 2.**
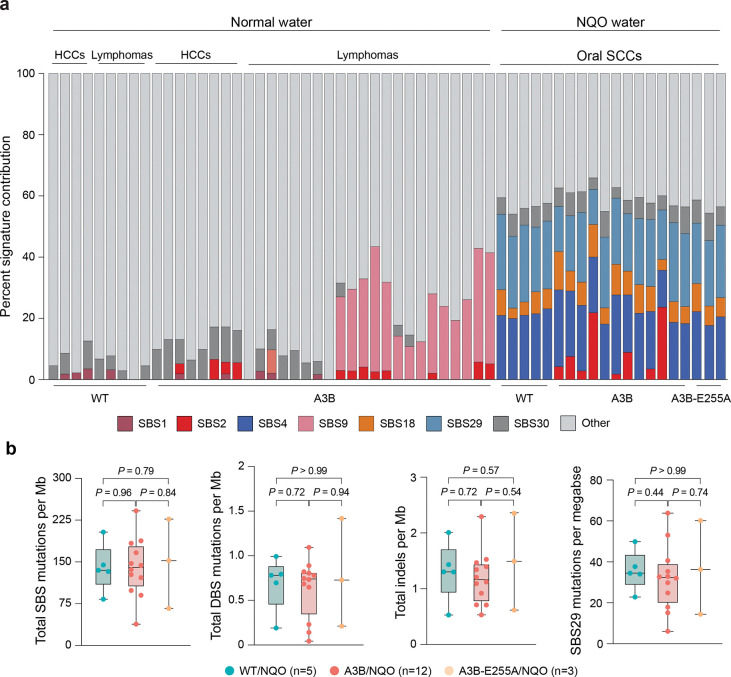
Additional data on mutations in tumors. (**a**) An alternative depiction of the WGS data in Fig. 2a. Here, the percentage of each SBS mutational signature is illustrated, in comparison to Fig. 2a that shows the frequencies of each mutational signature in mutations per Mb. The labeling scheme and the presentation order are identical. (**b**) Quantification of of the indicated classes of mutation in oral tumors from mice treated with NQO (SBS, single base substitutions; DBS, double base substitutions; Indels, insertion/deletions; tobacco/NQO-associated SBS29). Blue datapoints represent tumors from WT/NQO mice, red from A3B/NQO mice, and yellow from A3B-E255A/NQO mice. Boxplots are presented; the horizontal line within the boxes denotes the median and the boxes extend from the 25th to 75th percentiles (*P*-values, pairwise Mann-Whitney U tests).

**Figure 2.**
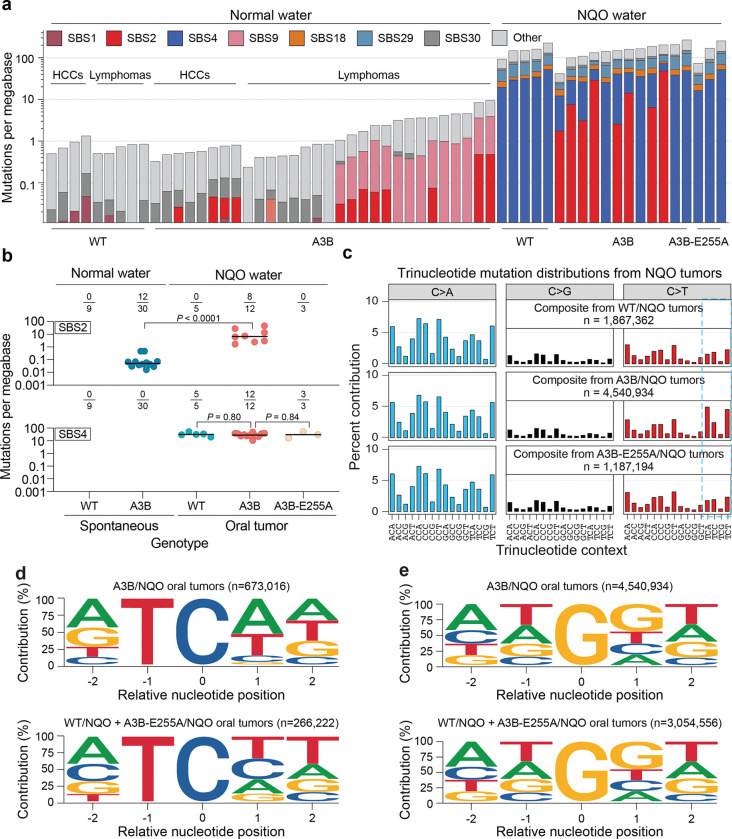
Synergistic increases in APOBEC signature mutations in A3B/NQO tumors. (**a**) Stacked histogram plots of SBS mutation loads in the indicated tumors from historic controls (left) and oral tumors from NQO treated animals (right). The most prevalent COSMIC SBS mutational signatures are color-coded with APOBEC/SBS2 in red and NQO/SBS4 in blue. The log10-scale Y-axis indicates mutation loads per megabase. (**b**) APOBEC/SBS2 (top) and NQO/SBS4 (bottom) mutation burdens in individual tumors from animals with the indicated genotypes and treatment conditions. The fractions above each dot plot report the total number of tumors with each mutational signature over the total number of tumors sequenced, and horizontal lines represent medians (*P*-values, pairwise Mann-Whitney U-tests). (**c**) Trinucleotide distributions of all C/G-to-A/T, -G/C, and -T/A mutations in tumors from NQO-treated WT, A3B, and A3B-E255A animals. N-values represent the combined total of C/G mutations from each experimental condition. APOBEC signature TCA and TCT motifs are preferentially mutated in A3B/NQO tumors (blue box). (**d**) Local context of TC-to-TT and -TG mutations in oral tumors from NQO-treated A3B animals (top) in comparison to NQO-treated control animals (WT and A3B-255A, bottom; n=total number of TC context mutations in each group). TC-context mutations in A3B/NQO tumors exhibit a prominent A3B mutation signature with a bias for A or G (purine) at the −2 position and for A or T (W) at the +1 position. (**e**) Local context of G-to-C and G-to-T (G-to-Y) mutations in oral tumors from NQO-treated A3B animals (top) in comparison to NQO-treated control animals (WT and A3B-255A, bottom; n=total number of G-to-Y mutations in each group). The two groups show nearly identical pentanucleotide contexts for NQO-induced G-to-Y mutations with a modest bias for guanine at +1 (and otherwise unbiased).

To ask if the observed tumorigenic synergy may be due to DNA level mutations, whole-genome sequencing (WGS) was done on oral tumors from NQO-treated WT, A3B, and A3B-E255A mice alongside matched tails as germline DNA references. This approach reveals massive SBS mutation burdens in tumors from NQO-treated animals (Fig. 2a; summarized in Extended Data Fig. 2a,b and **Supplementary Table 1**). Individual oral tumors show SBS mutation frequencies between 30 and 250 mutations per megabase, which equates to approximately 113,000–728,000 mutations per tumor and recapitulates mutation burdens observed in highly mutated human tumors^3,42,43^. All NQO-treated tumors contain SBS4 and SBS29 mutations attributable here to this DNA adducting agent and associated previously in human cancers with tobacco smoking and chewing, respectively^3,15,39^. Also, as expected, most tumors from A3B-expressing animals show an abundance of C-to-T mutations in TCA and TCT motifs (*i.e*., SBS2). For comparison, tumors isolated from naturally aged WT, A3B, and A3B-E255A animals from our prior studies^30^ do not harbor significant SBS4 or SBS29 mutations, and only a subset of tumors from A3B expressing animals exhibits SBS2 mutations (normal water groups in Fig. 2a, Extended Data Fig. 2a, and **Supplementary Table 1**).

Interestingly, in oral tumors with an APOBEC mutational signature from NQO-treated animals, the SBS2 mutation burden is an average of >100-fold higher than tumors from A3B animals provided with normal drinking water and aged naturally^30^ (SBS2 medians: 6.9 vs 0.05 mutations per megabase; *P*<0.0001 by Mann-Whitney U-test; Fig. 2b and summarized in **Supplementary Table 1**). This large increase is likely an underestimate because no oral tumors were available in control (normal water) groups for comparison at the same 32 week timepoint. Moreover, the previously obtained WT, A3B, and A3B-E255A tumors used for mutational comparisons here are from much older, naturally aged animals, where mutations have had much more time to accumulate (mostly >78 weeks)^30^.

In striking contrast, mutational burdens from tobacco-associated signature SBS4 are similarly high in all NQO-treated animals regardless of genotype, indicating that the observed SBS2 mutation increase from A3B constitutes a unique unidirectional synergy (SBS4 medians: 32 vs 28 mutations per megabase for A3B and WT mice, respectively; *P*=0.80 by Mann-Whitney U-test; Fig. 2b). In support of this relationship, there is an exclusive enrichment of C-to-T mutations in A3B-preferred RTCW motifs in A3B/NQO tumors, whereas NQO-induced C/G-to-A/T transversions occur at similar frequencies and local sequence contexts in all three genotypes (Fig. 2c,d,e). However, we note that NQO-induced G/C-to-T/A transversions have a modest bias toward GG dinucleotides (Fig. 2e). Curiously, 4/12 oral SCCs from A3B/NQO animals did not exhibit SBS2. At least one of these is due to somatic inactivation of the *A3B* minigene (remarkably, an E255A mutation) and the remainder have yet to be explained. Similar rates of SBS2 penetrance were observed in hepatocellular carcinomas and B lymphocyte tumors in our prior studies with naturally aged A3B animals^30^ further suggesting that A3B may impose a selective pressure that can be alleviated, in at least a subset of tumors, by its own mutational inactivation. Therefore, from hereonward our analyses focus on the 8 A3B/NQO tumors that exibit clear evidence for A3B activity in the form of SBS2 signature mutations.

### Coordinated pairs of APOBEC mutations (*didyma*) in NQO-treated tumors

**Extended Data Figure 3.**
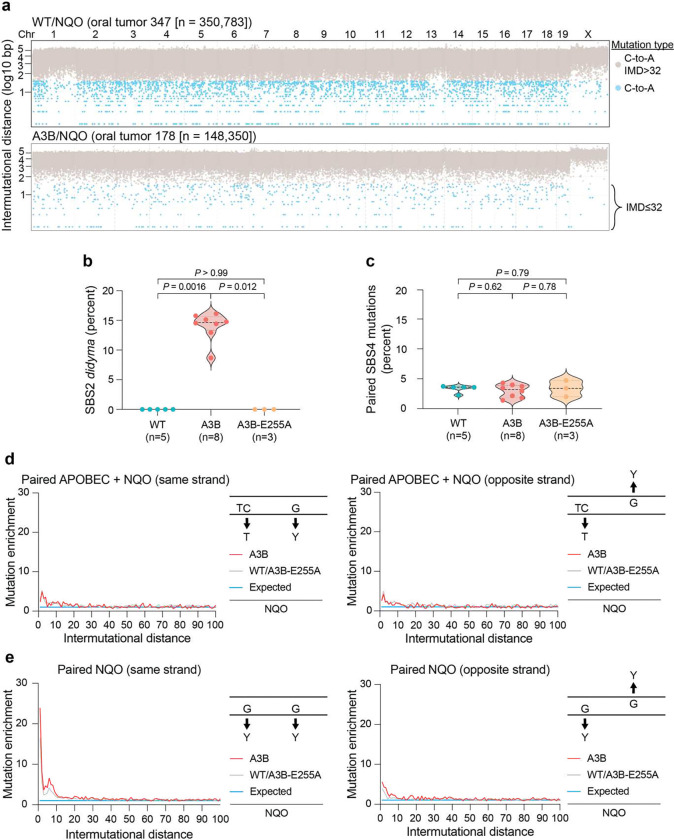
Additional data on paired mutations. (**a**) Rainfall plots depicting intermutation distances of all C/G-to-A/T mutations from representative WT/NQO and A3B/NQO tumors. Gray and light blue symbols represent SBS mutations with IMD >32 and ≤32, respectively. (**b-c**) Quantification of the percentage of SBS2 *didyma* (APOBEC) and the percentage of paired SBS4 mutations (NQO) with IMDs ≤32 in oral tumors from NQO-treated mice with the indicated genotypes. *n*-values for each group are indicated with the A3B/NQO group only including tumors with clear evidence for A3B function; *i.e*., the 8 tumors with a clear SBS2 mutational signature). The thick dashed horizontal lines represent medians, and the thinner dashed horizontal lines represent interquartile ranges (*P*-values, pairwise Mann-Whitney U-tests). (**d**,**e**) Observed vs expected enrichment values for the indicated mutational pairs over IMD distances 0 to 100 bp. The inset schematics illustrate the queried mutation pairs.

**Figure 3.**
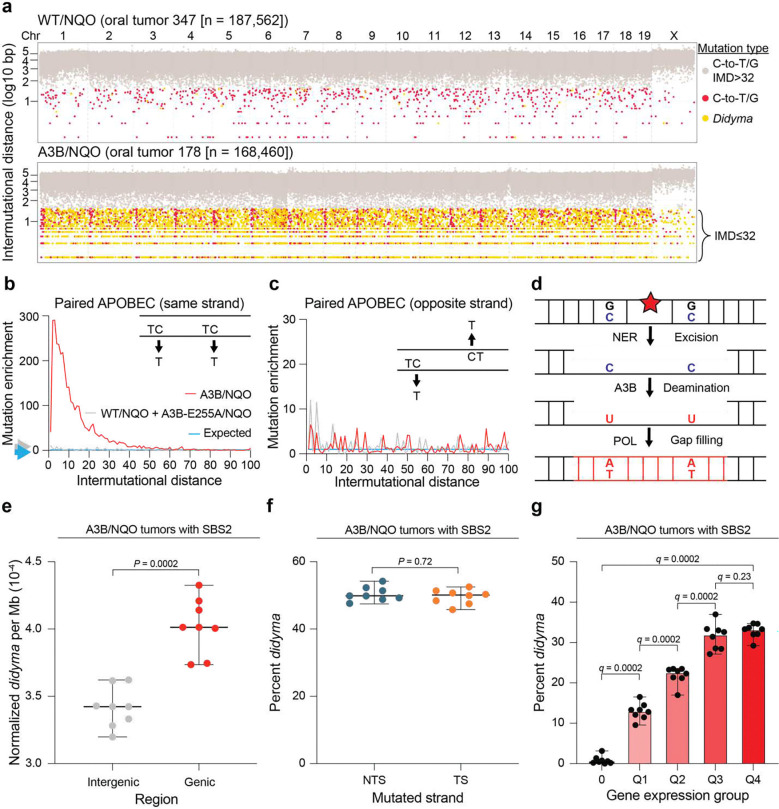
APOBEC *didyma* in NQO-treated tumors. (**a**) Rainfall plots depicting intermutation distances of all C-to-T and C-to-G mutations from representative WT/NQO and A3B/NQO tumors. Gray and colored symbols represent SBS mutations with IMD >32 and ≤32, respectively (red = two mutations where one or both is not in an APOBEC preferred trinucleotide motif; yellow = *didyma*, two mutations in APOBEC-preferred trinucleotide motifs TCA or TCT). (**b**) APOBEC *didyma* enrichment in A3B/NQO tumors (n=8, red line) vs the control groups combined (n=8, gray line). The expected enrichment based on simulations is also shown (blue line). The inset illustrates the queried mutational pairs. (**c**) An enrichment analysis similar to panel b except for pairs of APOBEC mutations on opposite strands (depicted in inset). (**d**) Schematic of the proposed molecular mechanism. Removal of a DNA adduct (red star) by NER creates a 24–32 nt ssDNA substrate for C-to-U deamination by A3B. Subsequent gap filling by a DNA polymerase (POL) immortalizes the U-lesions as paired APOBEC signature mutations (*didyma*). (**e**) A dot plot showing the normalized number of *didyma* per megabase occurring in intergenic versus genic regions of tumors from A3B/NQO animals. Horizontal lines represent medians and the whiskers 95% confidence intervals (n=8; *P*-value, Mann-Whitney U-test). (**f**) A dot plot showing the number of *didyma* occurring on the non-transcribed strand (NTS) or transcribed strand (TS) in A3B/NQO tumors. Horizontal lines represent medians and the whiskers 95% confidence intervals (n=8; *P*-value, Mann-Whitney U-test). (**g**) Percentage of genic *didyma* occurring in non-expressed genes or genes divided into quartiles based on expression levels in A3B/NQO tumors (Methods). Graph bars are medians and whiskers are 95% confidence intervals (n=8; *P*-values, pairwise Mann-Whitney U-tests).

We next constructed rainfall plots to visualize intermutation distances (IMDs) in representative tumors (Fig. 3a; Extended Data Fig. 3a). For instance, an oral tumor from an NQO-treated WT animal with 187,000 C-to-T/G mutations (613,000 total SBS mutations) shows that most of these mutations are separated by >1,000bp with few clustered events. In comparison, an oral tumor from a NQO-treated A3B animal with similar numbers of C-to-T/G mutations (168,000; 360,000 SBS mutations total) also shows a majority of dispersed mutations. However, this A3B/NQO tumor, as well as others from the same A3B/NQO condition, also exhibits a striking increase in a new type of paired mutation hereon called *didyma* (Greek for twins; Fig. 3a). *Didyma* are pairs of strand-coordinated APOBEC signature mutations occurring within a very short IMD ≤32 bp (colored yellow and forming a ladder-like arrangement 1 bp apart, 2 bp apart, 3 bp apart, *etc*., in Fig. 3a). *Didyma* comprise an average of 15% of all SBS2 mutations in A3B/NQO tumors, and they are non-existant in tumors from NQO-treated WT or A3B-E255A animals, which demonstrates that these unique mutational events are a direct result of A3B’s DNA deaminase activity (Extended Data Fig. 3b). APOBEC signature *didyma* are highly enriched in A3B/NQO tumors and, by contrast, non-strand-coordinated APOBEC signature mutations with the same ≤32 bp IMD are not (Fig. 3b,c). Moreover, although SBS4 mutations are much more abundant overall, paired NQO mutations with the same ≤32 bp IMD only comprise 3% of SBS4 mutations on average across all genotypes, and they are not enriched on the same or the opposing DNA strand (Extended Data Fig. 3c,d,e).

The observed one-way mutational synergy highlighted by *didyma* is most likely explained by a molecular mechanism in which the excision of bulky NQO lesions by nucleotide excision repair (NER) results in canonical 24–32 bp long ssDNA tracts, which are acutely susceptible to A3B-catalyzed deamination; the resulting ssDNA uracil lesions are subsequently immortalized as mutations by DNA polymerase-mediated gap-filling and strand ligation (*i.e*., error-free replacement of the excised DNA strand; Fig. 3d). This model is supported by elegant quantification of NER tract lengths in mammalian cells^44–46^, and by publications demonstrating that bulky NQO lesions are preferred substrates for this universally conserved DNA repair pathway^47–49^. Of course, short ssDNA segments created by NER are also substrates for single A3B-catalyzed C-to-U deamination events, in addition to *didyma*, but these mutational singlets are difficult to distinguish from other mechanisms including deamination of DNA replication and transcription (R-loop) intermediates. In further support of an NER-dependent mechanism, *didyma* occur preferentially in transcribed (genic) regions of the genome (Fig. 3e-f), where NER is known to be targeted through transcription-coupled DNA repair machinery^50,51^. Accordingly, *didyma* are rare in non-expressed genes and increase in frequency with level of gene expression, with the highest frequencies occurring in the most highly expressed genes (Fig. 3g).

### Notch pathway mutations and the overall genomic landscape in A3B/NQO tumors

**Extended Data Figure 4.**
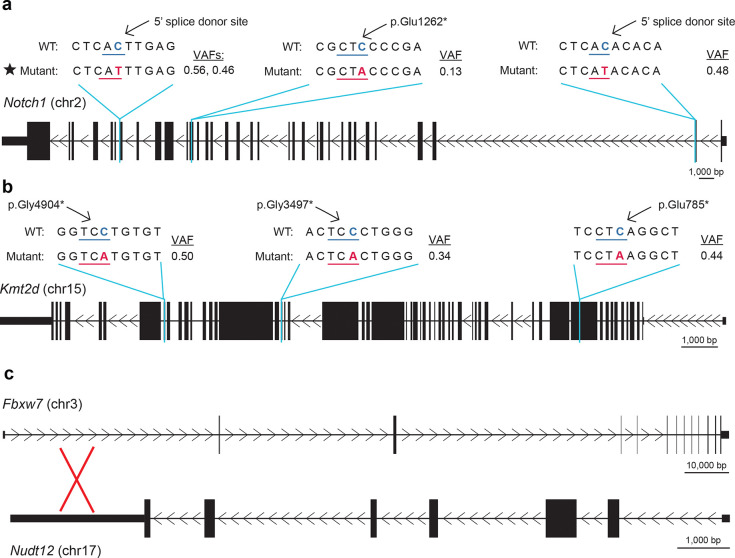
Individual mutations in Notch pathway genes. (**a-b**) Schematics of the *Notch1* and *Kmt2d* genes showing predicted high-impact mutations from Fig. 4a (scale = 1000 bp). The WT sequence is shown on top, aligned to each mutant sequence on the bottom. Variant allele frequencies (VAFs) are also shown to the right of each mutation. The black star highlights a splice site mutation that occurred in 2 independent tumors (also evidenced by different VAFs). (**c**) Schematic of the reciprocal translocation between *Fbxw7* and *Nudt12*. This translocation is predicted to disrupt *Fbxw7* expression by separating promoter region sequences from the majority of the gene body.

**Figure 4.**
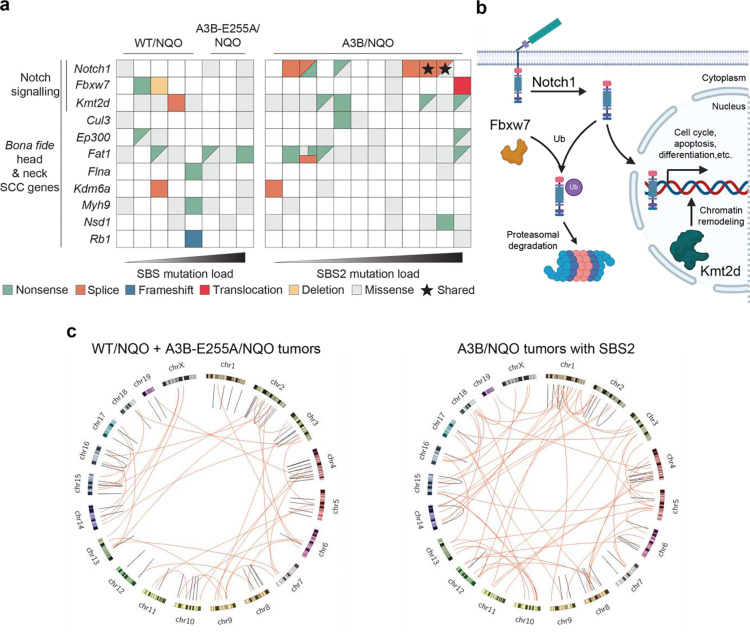
Notch signaling pathway alterations in A3B/NQO tumors. (**a**) Oncoprint representation of mutations in tumors from the indicated NQO-treated animals. The 11 genes listed here are *bona fide* human head & neck cancer genes. Each mutation type is indicated by a different color. The black star highlights a *Notch1* splice donor site mutation that occurred independently in two different tumors. (**b**) Schematic of the Notch signaling pathway. Proteins involved in Notch pathway signaling and disrupted in A3B/NQO oral tumors are labeled, including Notch1 itself, the ubiquitin ligase Fbxw7, and the methyltransferase Kmt2d. (**c**) Circos plots of structural variations occurring in control oral tumors (WT/NQO and A3B-E255A/NQO; n=8; left) and A3B/NQO oral tumors with SBS2 (n=8; right). Red lines represent translocations between the indicated chromosomes. Black lines represent intrachromosomal events (inversions, deletions, and duplications).

In human head & neck tumors, Notch signaling-associated genes are often mutated^42,52–54^. We therefore next asked whether this signal transduction pathway is altered in the oral tumors from NQO-treated A3B expressing animals. Intriguingly, *Notch1* had acquired high-impact mutations in 6/12 A3B/NQO tumors, including a c.5354+1G>A splice-site mutation in two independent tumors (Fig. 4a, Extended Data Fig. 4a). High-impact changes include nonsense and splice-site mutations predicted to be loss-of-function alleles. WT/NQO and A3B-E255A/NQO tumor groups exhibit fewer *Notch1* mutations and these are all missense mutations predicted to be low impact. *Notch1* inactivation can reduce epithelial differentiation, promote proliferation, and facilitate DNA damage accumulation in squamous cell carcinomas^55,56^. The A3B/NQO tumor with the highest SBS2 mutation burden has normal *Notch1* but has acquired somatic mutations in two Notch-pathway associated genes, a translocation involving *Fbxw7* and a nonsense mutation in *Kmt2d* (Fig. 4a, Extended Data Fig. 4b). Inactivation of *Kmt2d*, which encodes a histone-modifying protein, can repress Notch target gene expression and thereby similarly reduce differentiation^54^. A schematic depicts how these Notch pathway alterations may promote tumor cell growth (Fig. 4b).

As human head & neck tumors often also manifest larger-scale mutational events in addition to SBS mutations^42^, we also quantified indels and larger-scale chromosomal aberrations. These analyses suggest more structural variations in tumors with significant SBS2 from A3B/NQO animals in comparison to the WT/NQO or A3B-E255A/NQO combined (n=8 and n=8, respectively; Fig. 4c). This difference in structural variations is consistent with the occurrence of higher γ-H2AX levels in A3B/NQO tumors and with the likelihood that a subset of A3B-catalyzed DNA uracils can be processed into single- and double-stranded DNA breaks, intermediates in genetic recombination and chromosomal instability known to precipitate structural variation. At least one structural variation in A3B/NQO tumors, the reciprocal translocation between *Fbxw7* and *Nudt12*, may impact the Notch signalling pathway (Extended Data Fig. 4c). Taken together, these genomic analyses indicate that both SBS mutations and structural variations contribute to the observed synergistic increase in oral carcinomas.

### APOBEC signature mutations are elevated in tumors from smokers

**Figure 5.**
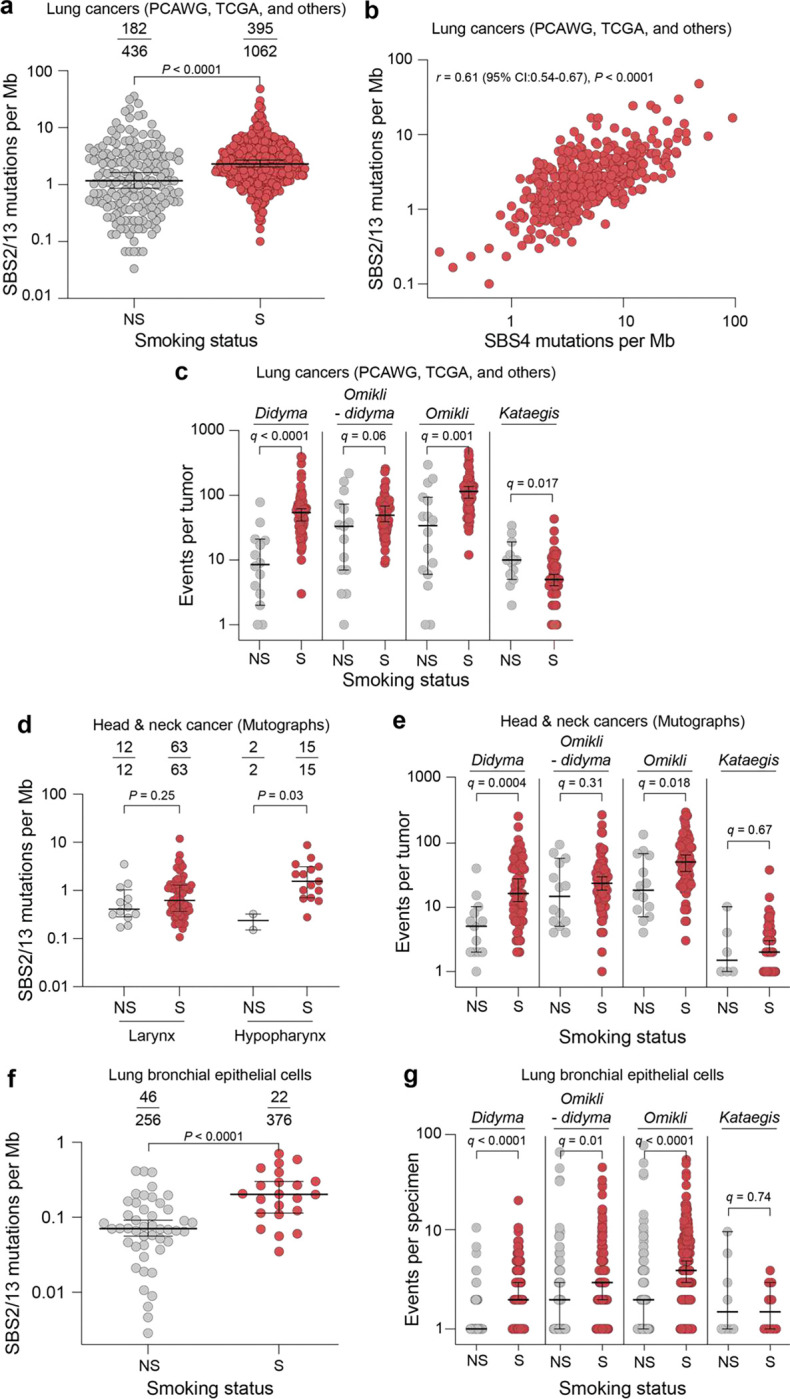
Elevated APOBEC signature mutations and *didyma* in tumors and normal tissues from smokers. (**a**) Quantification of APOBEC signature mutation loads (SBS2 and SBS13) in lung adenocarcinomas and SCCs from non-smokers (NS) and smokers (S). The fractions report the total number of tumors with an APOBEC mutational signature over the total number analyzed. The horizontal lines and whiskers represent medians and 95% confidence intervals (*P*-value, Mann-Whitney U-test). (**b**) A dot plot showing the direct relationship between APOBEC SBS2/13 mutations and tobacco smoking-associated SBS4 (n=395; *P*- and *r*-values, Spearman correlations). (**c**) Quantification of APOBEC-associated mutational events in lung tumors from smokers and non-smokers (*didyma*, *omikli* minus *didyma*, all *omikli*, and *kataegis*). The horizontal lines and whiskers represent medians and 95% confidence intervals, respectively (*q*-values, Mann-Whitney U-tests with Benjamini-Hochberg correction). (**d**) Quantification of APOBEC signature mutation loads in head & neck larynx and hypopharynx tumors from non-smokers and smokers. The horizontal lines and whiskers represent medians and 95% confidence intervals (*P*-values, Mann-Whitney U-tests). The fractions report the total number of tumors with an APOBEC mutational signature over the total number analyzed. (**e**) Quantification of APOBEC-associated mutational events in head & neck larynx and hypopharynx tumors from non-smokers and smokers (*didyma*, *omikli* minus *didyma*, all *omikli*, and *kataegis*). The horizontal lines and whiskers represent medians and 95% confidence intervals (*q*-values, Mann-Whitney U-tests with Benjamini-Hochberg correction). (**f**) Quantification of APOBEC signature mutation loads in pathologically normal lung bronchial epithelial specimens from non-smokers and smokers. The horizontal lines and whiskers represent medians and 95% confidence intervals (*P*-values, Mann-Whitney U-tests). The fractions report the total number of tumors with an APOBEC mutational signature over the total number analyzed. (**g**) Quantification of APOBEC-associated mutational events in normal lung bronchial epithelial tissue from non-smokers and smokers (*didyma*, *omikli* minus *didyma*, all *omikli*, and *kataegis*). The horizontal lines and whiskers represent medians and 95% confidence intervals (*q*-values, Mann-Whitney U-tests with Benjamini-Hochberg correction).

As cigarette smoke contains multiple DNA adducting carcinogens including benzo[*a*]pyrene and *N’*-nitrosonornicotine^2^, a major prediction from our A3B/NQO experiments in mice is that APOBEC-generated mutational signatures, SBS2 and SBS13, ought to be elevated in smokers compared to non-smokers (S and NS, respectively). To test this hypothesis, we analyzed a large dataset comprising 1,498 lung adeno and squamous cell carcinomas sourced from The Cancer Genome Atlas Program (TCGA), Pancancer Analysis of Whole Genomes (PCAWG), and other publicly available databases^3,57–61^. Data from 265 head & neck tumors^62^ were also analyzed to extend results to a second cancer type. First, we found that the average burden of APOBEC signature mutations is nearly double in lung cancers from smokers compared to non-smokers (median: 2.3 vs 1.2 mutations per megabase, respectively; *P*<0.0001 by Mann-Whitney U-test; Fig. 5a). Moreover, a remarkably strong positive association is evident between the frequency of mutational events attributable to APOBEC (SBS2+SBS13) and that attributable to smoking (SBS4; r=0.61, 95% CI=0.54–0.67, *P*<0.0001, Spearman’s correlation; Fig. 5b).

We next evaluated whether tobacco smoke carcinogens are associated with APOBEC mutation clusters including *didyma* in lung cancer. This analysis indicates that there are large increases in *didyma* in smokers compared to non-smokers (median: 54 vs 8.5 *didyma* per sample, respectively; *q*<0.0001 by Mann-Whitney U-test with Benjamini-Hochberg correction; Fig. 5c). There is also a similarly large increase in *omikli*, but not in *kataegis*. A probable explanation for this is that many *didyma* may actually be misclassified as *omikli* events, which were defined originally as a broader category of 2–3 clustered APOBEC mutations associated with mismatch repair^37^. This interpretation is supported by sub-analyses where *didyma* are subtracted from *omikli* and the significance of the latter mutational events diminishes substantially (compare results and statistics for *omikli* versus *omikli* minus *didyma* in Fig. 5c). *Kataegis* involves 4 or more strand-coordinated mutations and is therefore unlikely to occur within the short stretch of ≤32bp nucleotides exposed during NER. Accordingly, *kataegis* events appear at similar levels in smokers versus non-smokers (Fig. 5c).

In head & neck tumors, a strong 7-fold increase in APOBEC mutation density is apparent in hypopharyngeal tumors from smokers vs non-smokers but not in other tumor types of the head & neck (median: 1.5 vs 0.2 mutations per megabase for SBS2+SBS13, respectively; *P*=0.03 by Mann-Whitney U-test; Fig. 5d). Furthermore, we see a notable increase in *didyma* in tumors from the hypopharynx and larynx of smokers, whereas the same enrichment is not observed for *kataegis* or *omikli* after subtracting *didyma* (median: 16 vs 5 *didyma* per sample, respectively; *q*=0.0004 by Mann-Whitney U-test with with Benjamini-Hochberg correction; Fig. 5e). The observed synergy might be restricted to certain head & neck tumor sites because, like lung tissue, the hypopharynx is exposed after each inhalation for longer periods of time to mutagens from cigarette smoke^62^. Finally, to determine whether this mutational synergy can occur prior to tumor formation, we analyzed whole-genome sequencing data from 632 normal human bronchial epithelial specimens^63^. These results show that non-neoplastic lung cells from smokers have nearly 3-fold higher burdens of APOBEC dispersed mutational events as well as elevated *didyma* (median of 0.2 and 0.07 mutations per Mb, respectively; P<0.0001 by Mann-Whitney U-test; Fig. 5f; median of 2 vs 1 *didyma* in lung tissues from smokers and non-smokers, respectively; Fig. 5g). Taken together, these results indicate that these two very different mutational agents, tobacco smoke mutagens and APOBEC3 enzymes, can also combine synergistically in phenotypically normal lung epithelial cells.

## DISCUSSION

DNA damage and mutation processes are generally considered independent, with mutational outcomes accumulating additively over time^9,16,17^. Here, we employ mice as a biological *tabula rasa* to investigate oral tumorigenesis driven by mutations from the endogenous mutagen, human A3B, and the exogenous tobacco surrogate, NQO. These studies reveal an unexpected unidirectional synergy between these two processes. Specifically, the frequencies of A3B-inflicted SBS2 mutational events increase synergistically in NQO-treated animals, whereas the frequencies of NQO-induced SBS4 are similar under all conditions regardless of A3B status. This one-way mutational synergy is fully dependent on the deaminase activity of the A3B, as evidenced by a complete lack of SBS2 mutations in otherwise isogenic animals expressing an E255A catalytic mutant protein. Whole-genome DNA sequencing also demonstrates large numbers of pairs of A3B signature mutations with remarkably short IMDs ≤32 bp. These focused pairs of mutations are reminiscent of twins and here named *didyma* (a single pair is a *didymos*). A3B signature *didyma* are strand-coordinated and explained mechanistically by a model in which bulky lesions, such as NQO-guanine adducts, are excised by NER, and the resulting exposed single-stranded DNA tract is deaminated by A3B before being converted back into a protected duplex by DNA polymerase-mediated gap-filling (Fig. 2g).

These results are directly relevant to lung and head & neck cancers and also likely to be broadly relevant to any cell exposed to a DNA adducting agent requiring resolution by NER. First, we find strongly elevated levels of APOBEC signature SBS2 and SBS13 mutations in smokers compared to non-smokers (Fig. 4a). This difference has been noted in prior studies but without mechanistic explanation^4,40^. Moreover, here we uniquely uncover a strong positive association between APOBEC mutation loads (SBS2/13) and smoking-associated mutation loads (SBS4) in whole-genome and whole-exome sequenced lung cancers from TCGA, PCAWG, and other publicly available sources^57–60^ (Fig. 5b). Second, we demonstrate that large percentages of APOBEC signature SBS2 and SBS13 mutations in smokers manifest as *didyma* in lung tumors from smokers but not in non-smokers (Fig. 3a,b and Extended Data Fig. 3b). Because NER is a universally conserved DNA repair mechanism^50,64,65^, these hallmark *didyma* are almost certainly attributable to a mechanism in which two processive APOBEC deamination events are inflicted in ssDNA NER intermediates prior to gap-filling by DNA synthesis and ligation (Fig. 3d). It is further notable that *didyma* (2 strand-coordinated APOBEC signature mutations with an IMD ≤32) are distinct mechanistically from mismatch repair-associated *omikli* and DNA recombination- and transcription (R-loop)-associated *kataegis* (respectively with 2–3 or ≥4 strand-coordinated APOBEC signature mutations, respectively)^31,35,37^. Moreover, the majority of APOBEC *omikli* in lung tumors from smokers appear to be *didyma* through the mechanism described here (Fig. 5c). Third, head/neck cancer data are used to show that APOBEC signature mutations and *didyma* are exacerbated in smokers in comparison to non-smokers (Fig. 5d,e). Last, but not least, we discover similar APOBEC mutational signature and *didyma* enrichments in phenotypically normal lung broncheolar specimens from smokers in comparison to non-smokers, indicating that this one-way mutational synergy can also occur prior to visible cancer development (Fig. 5f,g).

Given the universal nature of NER, it is tempting to speculate that *didyma* will be found anywhere cellular DNA is damaged by bulky chemical adducts and, importantly, A3B is expressed constitutively or induced transiently. For instance, common mutagens that generate bulky lesion agents include aristolochic acid from herbal remedies and dibenz[a,h]anthracene from fuel combustion, which are associated with kidney and lung cancers, respectively^66,67^. Even classical NER lesions, pyrimidine dimers and 6–4 photoproducts from UV light exposure, might trigger synergistic increases in APOBEC mutagenesis in a subset of skin cancers. Moreover, frequently used chemotherapeutics including platinum-based therapies such as cisplatin, carboplatin, and oxaliplatin create intra- and interstand crosslinks that require processing by NER to correct. Further exploration of potential synergies between these therapeutics and A3B could help to stratify patients receiving platinum-based compounds into differential treatment response groups.

The *in vivo* studies conducted here with NQO treatment of human A3B expressing mice reveal a strong synergy at the pathological level through tumor formation and at the molecular level through mutational signature. This is a striking one-way synergy in which A3B SBS2 mutational events increase synergistically but NQO SBS4 events do not. Analogous experiments have yet to be conducted with other cancer-associated APOBEC family members including A3A, APOBEC1, and AID but, given the processive nature of these enzymes^68,69^, the fact that most family members preferentially deaminate ssDNA substrates^28,31,68^, and the high prevelance of APOBEC signature mutations in the majority of human cancer types^3,7,23,70^, it is likely that the example detailed here will constitute the first of many studies on mutational synergies between A3B, related deaminase family members, and a wide variety of exogenous DNA mutagens and carcinogens.

## Methods

### Animal model maintenance

Mice were housed at the University of Minnesota Twin Cities and University of Texas Health San Antonio animal facilities in specific pathogen-free conditions at an ambient temperature of 24°C under a standard 12h light/dark cycle. Standard breeding and husbandry for cancer studies, as well as NQO treatments, were included in protocols reviewed and approved by Institutional Animal Care and Use Committees (IACUC protocols 2201–39748A and 20220024AR, respectively).

B6*.Rosa26::CAG-LSL-A3Bi* mice and B6.*Rosa26::CAG-LSL-A3Bi-E255A* mice have been described^30^ and deposited in Jackson Laboratory (Jax #038176 and #038177, respectively). These animals were crossed with B6.C-Tg(CMV-cre)1Cgn/J mice (Jax #006054) to excise the transcription STOP cassette (*i.e*., reduce *loxP-STOP-loxP* to a single *loxP* site by Cre-mediated recombination). Subsequent crosses with WT C57BL/6 animals yielded the experimental cohorts described here including WT littermates for the control cohort. Mice were genotyped for the *Rosa26*, *Rosa26::CAG-L-A3Bi*, and *Rosa26::CAG-L-A3Bi-*E255A alleles using the following PCR conditions: 1) 95°C for 30 seconds; 2) 68°C for 30 seconds; 3) 72°C for 1 minute; 4) repeat steps 2 – 4 11 times; 5) 95°C for 30 seconds; 6) 68°C for 30 seconds; 7) 72°C for 1 minute; 8) repeat cycles 5 – 7 25 times. Primers are as follows:

*Rosa26* forward: 5’-AGCACTTGCTCTCCCAAAGTC

*Rosa26* reverse: 5’-CACCTGTTCAATTCCCCTGC

*CAG-L-A3Bi* forward: 5’-CGTGCTGGTTATTGTGCTGT

*CAG-L-A3Bi* reverse: 5’-TCCGCTCCATCGGATTTCTG

Mice were genotyped in parallel for Cre and *Interleukin 2* (housekeeping control) using the following PCR conditions: 1) 94°C for 3 minutes; 2) 94°C for 30 seconds; 3) 51.7°C for 1 minute; 4) 72°C for 1 minute; 5) repeat steps 2 – 4 35 times; 5) 72°C for 3 minutes. Primers are as follows:

*Cre* forward: 5’- GCGGTCTGGCAGTAAAAACTATC

*Cre* reverse: 5’- GTGAAACAGCATTGCTGTCACTT

*Interleukin-2* forward: 5’- CTAGGCCACAGAATTGAAAGATCT

*Interleukin-2* reverse: 5’- GTAGGTGGAAATTCTAGCATCATCC

Master mixes for all reactions consisted of final concentrations of 1x Taq buffer (Denville Scientific), 1 mM dNTPs (Thermo Scientific), 0.3 units of Taq polymerase (Thermo Scientific), 1 μM of each primer (Integrated DNA Technologies), and 25 ng of genomic DNA.

### Oral tumor induction and analysis

Animals of each genotype were enrolled randomly at 8 weeks of age for treatment with NQO water. 4-NQO powder (Sigma-Aldrich) was dissolved in 100% DMSO to create a 5 mg/mL stock solution, which was subsequently diluted in water to 50 μg/mL for administration to animals. NQO water was provided continuously from week 9 to week 24, and all animals were switched to normal water for weeks 25–32. At 32 weeks of age, animals were sacrificed by CO_2_ asphixiation, subjected to necropsy and pathological examination, and surgically dissected for collection of tongue, oral soft tissues, esophagus, duodenal tissues, and tails. Half of each tissue was used for genomic DNA preparation and the remainder was fixed overnight in 10% buffered formalin (10% formalin, 90% distilled water, 5 mM Na_2_HPO_4_). Tongues were trisected, embedded in paraffin blocks, stained using hematoxylin & eosin (H&E) as below, and subsequently analyzed by a board-certified oral and maxillofacial pathologist under fully blinded conditions. Oral lesions were quantified by considering clinically or microscopically distinct exophytic papillary high-grade epithelial dysplasias and invasive SCCs in the oral cavity only, including tumors of the tongue, buccal, or labial mucosa. In addition to the oral cavity, the esophagus and duodenum of each animal were also harvested and histopathologically examined for epithelial lesions. As anticipated, lesions were confined to the oral cavity and the esophagus. Lesion thickness and depth of invasion were measured using the Keyence BZ-X800 Analyzer software. Lesion thickness was determined by measuring the distance from the apical surface of the epithelium (keratin layer) to the basal cell layer. Depth of invasion was quantified by measuring the distance from the basal cell layer of normal adjacent-to-tumor epithelium to the deepest edge of invading carcinoma nests.

### Hematoxylin & eosin (H&E) staining

Formalin-fixed paraffin-embedded (FFPE) tissues were sectioned into 4 μm slices and mounted onto positively charged adhesive glass slides. Slides were subsequently baked at 60°C for 20 min, washed using xylene 3 times for 5 min, immersed in a series of graded alcohols (100% × 2, 95% × 1, and 80% × 1) for 2 min each, and rinsed in tap water for 5 min for deparaffinization and rehydration. Slides were stained with hematoxylin for 5 min, rinsed in tap water for 30 seconds, subsequently submerged in an acid solution and 60 seconds in ammonia water. Slides were then washed with tap water for 10 min, immersed in 80% ethanol for 1 min, counterstained with eosin for 1 min, dehydrated in graded alcohols (as above but inverted in increasing concentrations) followed by xylene, and coverslipped with Cytoseal (Thermo Scientific). High-resolution digital images were acquired using a Keyence all-in-one fluorescence microscope BZ-X800.

### Immunohistochemical staining

Immunohistochemistry (IHC) was done as described^30,71,72^. FFPE tissues were sectioned into 4 μm slices and mounted on positively charged adhesive slides. Tissue slices were baked at 65°C for 20 min, then immersed in CitriSolv (Decon Labs) for 5 min each followed by graded alcohol washes as in the precedent section and a 5 min tap water rinse for deparaffinization and rehydration. 1x Reveal Decloaker (BioCare Medical) at pH 6.0 was used for epitope retrival, steaming encased slides for 35 min with a subsequent 30 min off the steamer. Slides were then rinsed with running tap water for 5 min followed by submersion in Tris-buffered saline with 0.1% Tween 20 (TBST) for 5 min. Endogenous peroxidase activity was stifled with a 10 min soak in 3% H_2_O_2_ diluted in TBST and successive tap water rinse for 5 min. Nonspecific binding was blocked using a 15 min soak in Background Sniper (BioCare Medical). Ensuing primary antibody incubation was carried out at 4°C overnight using primary antibody diluted in 10% Background Sniper in TBST. Primary antibodies used for detection were directed against A3B (5210-87-13^41^) at a 1:500 dilution and γ-H2AX Ser139 (Cell Signaling cat# 9718) at a 1:200 dilution. Directly after overnight incubation, samples were rinsed with TBST for 5 min and then incubated using Novolink Polymer (Leica Biosystems) for 30 min to visualize the rabbit IgG primary antibodies. Signal was developed by application of the Novolink DAB substrate kit (Leica Biosystems) for 5 min, rinsed with tap water for 5 min, and counterstained with Mayer’s hematoxylin solution (Electron Microscopy Sciences) for 10 min. Finally, slides were washed with tap water for 10 minutes and dehydrated in graded alcohols and CitriSolv, then cover-slipped with permount mounting media (Thermo Scientific).

### DNA extraction

Genomic DNA was extracted from fresh frozen oral tumors and matched normal tails using the DNeasy Blood and Tissue Kit (Qiagen). Tissues were homogenized using Qiashredder columns (Qiagen) and genomic DNA was prepared according to the manufacturer’s instructions.

### Whole-genome sequencing

100 ng genomic DNA from each oral tumor was used for WGS library preparation using the NEBNext Ultra II FS DNA Library Prep Kit for Illumina (New England Biolabs). The genomic DNA was broken with an enzymatic fragmentation reaction that simultaneously repairs ends and adds dA-tails, and then each reaction was subsequently cleaned up using KAPA Pure Beads to ensure a uniform library insert size. The library was then amplified using 5 PCR cycles: 1) 98°C for 30 seconds; 2) 98°C for 10 seconds; 3) 65°C for 75 seconds; 4) repeat steps 2 and 3 thrice; 5) 65°C for 5 minutes. The final DNA sequencing library was cleaned up using KAPA Pure Beads and quantified using an Invitrogen Qubit 3 Fluorometer and an Agilent Tape Station. Libraries were normalized to 10 nM and pooled at equimolar concentrations and sequencing on an Illumina NovaSeq 6000 Sequencing System to approximately 30x coverage with 150 bp paired end sequencing. Following the sequencing run, sample demultiplexing was performed on instrument to generate FASTQ files for each sample.

### Somatic mutation calling

Mouse whole-genome sequencing paired reads were trimmed with Trimmomatic v0.40-rc1^73^ and then aligned to the mouse genome mm10 using BWA v0.7.17-r1188^74^. PCR duplicates were removed by MarkDuplicates module of GATK v4.2.6.1^75^. Reads were locally realigned around indels using RealignerTargetCreator and the IndelRealigner module of GATK3 v3.8–1-0-gf15c1c3ef. Single base substitutions and small indels were called relative to the matched normal tissues individually using Mutect2 module of GATK v4.2.6.1^76^, MUSE v2.0^77^, Strelka2^78^, and VarScan v2.4.6^79^. Single base substitutions and small indels identified by ≥ two callers were accepted as true mutations to reduce false positives. These candidate mutations were additionally filtered by requiring at least 3 reads supporting the mutation, a minimum of 10 reads at each variant site, and a variant allele frequency (VAF) over 0.05. These filtered calls were used for downstream analyses below. SnpEff was used to determine which SBS mutations and indels resulted in high- or moderate-impact mutations in genes^80^.

### Structural variation calling

Somatic structural variations were detected by comparing tumor to matched normal tissues and implementing four independent programs including: Manta with a minimum somatic score of 40^81^; SvABA v1.1.0^82^; Delly with PRECISE and PASS status ^83^; and Gridss v2.13.2 with a quality score higher than 500^84^. Structural variations that were observed within 100 bp of each other in at least two of these algorithms were used for downstream analyses. Circos plots of structural variations were generated by Galactic Circos^85^.

### Mutational signature analysis

Mutational landscapes from mouse tumors were plotted using MutationalPatterns R package^86^. Known signatures from COSMICv3.4 were assigned utilizing a two pass non-negative least squares fitting where a user defined cut-off (0.015 in this study) is applied to remove low contribution signatures after first pass using package (https://github.com/temizna/SigAssignR). Mutational signatures in humans were assigned using SigProfilerAssignment v0.1.9 to decompose the SBS mutational signatures extracted in the original publications^60,62^ into known signatures present in COSMICv3.4^6^. For normalization of mutation burdens between different samples, we assume 2,723 megabases (Mb) to be sequenced from mouse whole genomes, 2,800 Mb for human whole genomes, and 30 Mb sequenced for human whole exomes. For human lung cancer datasets, a large portion lacked clinical metadata and therefore smoking status was not annotated. This challenge was overcome using SBS4 to separate data from smokers (S) and non-smokers (NS).

### Mutational context assignment

Pentanucleotide contexts were extracted using MutationalPatterns^86^. Genome wide distributions of pentanucleotides were calculated using mm10 genome. The mutation frequencies of each pentanucleotide context were adjusted using the genome wide distributions of the pentanucleotides.

### IMD simulation and paired mutation calculations

Clustered mutations were extracted from detected somatic mutations of each individual sample as described^87^. Briefly, high confidence somatic mutations called from 2 of 4 different mutation callers were combined, and SigProfilerSimulator v1.1.6^88^ was used to simulate a background trinucleotide mutational signatures on every chromosome with strand asymmetry and genic location taken into consideration. SigProfilerClusters v1.1.2^87^ was used to determine sample-dependent intermutational distance (IMD), capturing 90% of mutations below it as unlikely to occur by chance (q-value < 0.01). Genome-wide imbalanced mutation distributions were further corrected on mutations by applying an additional regional IMD cut-off based on real and simulated mutation numbers within a 1 Mb size sliding genomic window. Maximum VAF difference with a cut-off of 0.1 was used to finalize clustered mutations, ensuring that clustered mutations events occurred in same cells.

Paired mutations were defined based on mutational context. For A3B *didyma*, this was defined as C-to-T and C-to-G mutations in a TC context. For NQO, this was defined as G-to-T and G-to-C mutations (except for G-to-C mutations in a GA context to avoid conflation with *didyma*). Mutations were classified as occurring on either the same strand or opposite strands based on the strand orientation of the reference nucleotides. Mutation enrichment was defined as the number of observed mutations divided by the number of simulated mutations. Here, the observed paired mutation were those that occur in the collected tumors. Simulated paired mutations were derived from simulated mutations occurring at random across each chromosome in the genome as described above. One hundred rounds of iterative simulation were performed, and the average of each type of simulated mutation pair was used for each enrichment analysis. For every intermutational distance, the number of observed mutations was divided by the number of simulated mutations, and then values were averaged for all A3B/NQO samples or combined WT/NQO and A3B-E255A/NQO samples.

### Transcriptional strand analysis

Paired APOBEC signature mutations within 32 nucleotides of each other were analyzed for strand bias using SigProfilerExtractor v1.1.24^60^ and SigProfilerTopography v1.0.86^89^.

### Mutation and gene expression analysis

Transcriptomes of murine SCCs from NQO-treated and of normal tongue samples were obtained from a prior study^90^. Transcript expression was normalized with DESeq2^91^ and binned into quartiles or a fifth group for no expression. Mutations from mouse oral tumors were then annotated as occurring in labelled gene regions including exons and introns or intergenic regions based on the GENCODE M10 (GRCm38.p4) genome assembly. To compare the preference for *didyma* to occur in these regions, mutations were normalized according to the number of mutations per megabase (where genic regions include exons and introns – 1,139 Mb – and the rest of the genome is intergenic – 1,584 Mb). Mutation data were then concatenated with published transcriptome data^90^ to determine if *didyma* occur in transcribed regions and associate with transcript levels.

### Clustered mutation analysis

Sample-dependent IMDs were extrapolated from each sample (mouse and human) using SigProfilerSimulator v1.1.6^88^ and SigProfilerClusters v1.1.2^87^. Only SBS mutations were included in these analyses, all indels were discarded. IMDs were calculated as the number of nucleotides separating consecutive mutations (*e.g*., TCC = 1 IMD; TCTC = 2 IMD). For human samples, APOBEC-specific mutation clusters were extracted by assessing all mutations that fall within the sample-dependent IMD and are exclusively strand-coordinated and entirely consist of APOBEC context mutations in a T[C-to-T/G]W context. They were further divided into two groups: *omikli* for events with two to three mutations and at least one IMD greater than 1; and *kataegis* for events with four or more mutations including at least one IMD greater than 1. *Didyma* events were extracted by considering all strand-coordinated APOBEC-context mutations that fall within 32 nucleotides of each other. *Omikli* – *didyma* were defined as *omikli* events that have IMDs > 32 nucleotides.

### Statistical analyses

Comparisons were conducted using non-parametric statistical tests, namely two-tailed Mann-Whitney U-tests for comparisons, Spearman’s correlation for association, and Poisson regression as noted for comparison across two independent experiments. *P*-values were adjusted for multiple comparisons using Benjamini-Hochberg correction methods within figures presenting 4 or more statistical tests and denoted as *q*-values. Details and statistical values are provided in each figure legend. Analyses were conducted using Prism 10.3.0 and SAS 9.4 (Cary, NC).

### Data availability

All murine tumor genomic DNA sequences reported here will be available in the Sequence Read Archive coincident with publication. Human lung cancer data were gathered from the PCAWG consortium and others, which are publicly available, with all somatic mutation data downloaded from ref.^59^. Similarly, somatic mutation data were publically available for bronchial epithelial tisssue^63^ and human head & neck cancers^92^, and these data sets were downloaded from the respective publications for new analyses here.

## Extended Data
